# Violent Behaviour and Emotional Intelligence in Physical Education: The Effects of an Intervention Programme

**DOI:** 10.3390/ejihpe14070124

**Published:** 2024-06-24

**Authors:** Manuel Ortiz-Franco, Félix Zurita-Ortega, Eduardo Melguizo-Ibáñez, Gabriel González-Valero, David Lindell-Postigo, José Luis Ubago-Jiménez

**Affiliations:** 1 La Inmaculada Teacher Training Centre, 18016 Granada, Spain; 2 Department of Didactics of Musical, Plastic and Corporal Expression, University of Granada, 18071 Granada, Spain; felixzo@ugr.es (F.Z.-O.);; 3 Department of Physical Education, Sunland International School, 29580 Málaga, Spain

**Keywords:** secondary education, student violence, sport psychology, emotional intelligence, martial arts

## Abstract

Violent behaviour in the secondary education stage is a serious concern that comes from low emotional control. Judo is a sport that requires self-control and high emotional competence to mitigate aggressive behaviours. This research presents the objectives of analysing the correlations of different types of aggressive behaviours before an intervention program with those after said program, as well as study the effect of emotions on aggressive behaviours before and after the intervention program through multigroup structural equation modelling. A quasi-experimental study was planned. It used a pre-test–post-test design in a population of 139 secondary school students (M = 15.76; SD = 1.066). The instruments were an ad hoc questionnaire, the Schutte Self-Report Inventory and the Violent Behaviour at School Scale. The data show that the intervention decreased the correlations between different types of violent behaviours. The results show an increase in the effect of emotional intelligence on mitigating aggressive attitudes. The promotion and use of contact sports is necessary to prevent the emergence of aggressive behaviours within a school environment.

## 1. Introduction

Physical education plays a fundamental role in the educational and formative processes of students [1]. It enables young people to develop motor, cognitive and affective skills through situations based on play and sport under a playful and recreational background [2]. Taking advantage of the character that this discipline possesses, research on physical education has focused on the control and management of emotions, highlighting improvements in the emotional competencies of young people [1,2].

Emotional intelligence has acquired a high degree of relevance in different educational areas. It is defined as the competence to understand and recognise one’s own and other people’s emotions [3,4]. Discipline in physical education helps students improve their emotional states [1]. Through practicing physical activity, young people experience situations where the regulation of emotional states or the development of emotional self-awareness is required [1]. The proper control of emotions positively affects academic life, quality of life and self-control in the face of negative emotions [5].

If negative emotional states are sustained over time, they are likely to result in an aggressive or violent response [5]. Emotional intelligence acts as an element that helps prevent disruptive responses through self-control [5]. In a school environment, different types of aggressive behaviours are exhibited that do not necessarily require physical contact [6]. Specifically, Musitu et al. [6] point out that there are different forms or types of aggression, divided into overt or direct aggressive acts that involve direct confrontation between the victim and the aggressor and indirect relational aggressive acts that involve the aggressor not being directly involved [6]. Differences can also be found according to the purpose of the aggression [6]. Reactive aggression refers to different behaviours that involve defensive action in response to provocation [6]. Instrumental aggression is goal-oriented and pre-planned [6], and pure aggression refers to aggressive behaviours that are triggered without an aggressive stimulus [6]. Intervention studies have been carried out in the field of physical education. Through practicing physical activity, it has been possible to reduce the levels of violence in the adolescent population [7].

Regularly practicing sporting activities has been reported to have psychosocial benefits. Specifically, the psychosocial effects of fighting sports have been highlighted [8]. Judo is regarded as a combat sport and a martial art that initially built its philosophy around fighting under a eudaimonic way of life [9]. Regular Judo practice leads to greater emotional control and a reduction in aggressive and violent behaviour [7,9], but there is little research on how the implementation of a Judo intervention programme may influence these constructs. Using interventions focused on Judo, Montero-Carretero et al. [7] succeeded in reducing bullying and violent behaviour through a total of ten sessions. Broadening the field of research, the effectiveness of contact sport interventions to reduce disruptive behaviour has been demonstrated [7,10].

This research thus establishes the following research hypotheses:

**H.1.**  *The Judo-based intervention programme will decrease the relationship between different types of aggressive behaviour.*

**H.2.**  *The intervention will enhance the effect of emotional intelligence on different types of aggressive behaviour.*

The objectives are as follows:

**O.1.**  *To analyse the correlations of different types of violent behaviour before and after the intervention programme.*

**O.2.**  *To study the effect of emotional intelligence on aggressive behaviour before and after the intervention programme using structural equation modelling.*

## 2. Materials and Methods

### 2.1. Sample and Design

The intervention programme featured a single intervention group. Pre-programme and post-programme data were collected. The participants of this study were 139 secondary school students (Age = 15.76; SD = 1.066). The sample was homogeneous, as 70 were boys and 69 were girls. Due to the fact that the participants were minors, the educational centre contacted the student’s parents. Once the legal guardians were informed, they authorised their children to participate in this study.

### 2.2. Instruments

**Ad hoc socio-demographic questionnaire:** This was employed to collect socio-demographic variables. The first of these was the gender (male/female) and age of the participants.

**Schutte Self-Report Inventory (SSRI)** [11]: A version adapted into Spanish was used [12]. Emotional intelligence was assessed through four sub-variables: Emotional Perception, Self-Emotional Management, Heteroemotional Management and Emotional Use. The following reliability indices were obtained: Emotional Perception α = 0.893, Self-Emotional Management α = 0.888, Self-Emotional Management α = 0.867 and Emotional Utilization α = 0.852.

**Violent Behaviour at School Scale** [13]: The version adapted by Musitu et al. [6] was used. It consists of 25 items. The instrument assesses violent behaviour through overt aggression (the aggressor and the victim are present) and relational aggression (the aggressor remains anonymous). A value of α = 0.847 was obtained for overt aggression. A value of α = 0.758 was obtained for relational aggression.

### 2.3. Procedure

Before starting fieldwork, an ethics committee was formed (2966/CEIH/2022). The sections established in the Helsinki declaration were also followed to guarantee the safety of the participants at all times.

Once all the permissions related to research ethics had been obtained, the head teacher of the school and the PE teacher were contacted to explain the objectives of this research in detail. Once a favourable response was obtained, the different legal guardians of the students were contacted. An informative letter was sent to them informing them of the research objectives and explaining the intervention programme. Once the minors had given their consent, the research team contacted the participants.

The intervention programme was carried out by the school’s physical education teacher. It should be noted that the physical education teacher at the school was the one who taught all the sessions of the intervention programme. The teacher had information and experience related to the teaching of contact sports. He also had the rank of second dan in Judo. The rest of the research team provided support during the sessions to help them run smoothly. It should also be noted that the programme was carried out during physical education sessions; therefore, the entire intervention was duly contextualised within the Spanish educational regulations of secondary education.

The intervention lasted 24 sessions, with a total of 2 classes per week. The sessions lasted one hour. The time distribution of the sessions was organised as follows: warm-up and joint mobility (5 min); technique exercises, gripping, practice of falls, projection techniques and immobilisations (45 min); and return to calm and resolution of doubts and questions (10 min). Specifically, the intervention programme was divided into two clearly differentiated parts. The first was centred on the techniques related to ground Judo (Hon-Kesa-Gatame, Kami-Shiho-Gatame, Yoko-Shiho-Gatame and Tate-Shio-Gatame). The second part of the programme focused on the teaching and application of basic falls (Ushiro Ukemi, Hidari Yoko Ukemi, Migi Yoko Ukemi, Mae Ukemi and Mae Mawari Ukemi) together with Judo foot techniques (O-Soto-Gari and Uki Goshi).

All sessions were conducted on the school’s tatami mat. Before implementing the intervention programme, the installation was checked in order to ensure that it was in good condition. This was performed in order to ensure that the facility was in good condition and did not compromise the safety of the young people.

The distribution of the programme is presented below.

**Session 1 to Session 3:** Immobilisation games. The teaching style was based on problem solving, with the students taking the lead.

**Session 4 to session 10:** Judo floor exercises. During sessions 9 and 10, the students practised randori. This occurred in order for the students to apply sight techniques in combat situations.

**Session 11:** Exam of the immobilisation techniques. A checklist was used by the teacher for the evaluation.

Session 12 to Session 16: Falling games.

**Session 16 to Session 20:** Explanation of Judo foot techniques.

**Session 21 to Session 23:** Randori applying Judo foot techniques.

**Session 24:** Exam of Judo foot techniques. A checklist was used by the teacher for the assessment.

### 2.4. Data Analysis

The following section presents the analyses carried out for the data analysis.

IBM SPSS Statistics was used to analyse the distribution of the data. Specifically, version 25.0 of this programme was used. The Kolmogorov–Smirnov (K-S) test was used to analyse normality. The analysis showed a non-normal distribution; therefore, non-parametric tests were used. For correlational analysis, Spearman’s correlation coefficient was used. The significance level was set at *p* ≤ 0.01.

IBM SPSS Amos software was used to develop the theoretical model. Specifically, version 23.0 of this programme was used. Analysing the proposed theoretical model, it comprises eleven variables. Ten of them play an endogenous role. Only one is an exogenous variable. The endogenous variables are those that receive the effect of other variables [14]. Exogenous variables are those that exert an effect on the endogenous variables [14]. Due to the characteristics of the endogenous variables, the relationship between measurement reliability and the different indicators has been considered. The arrows in the model symbolise the direction and direction of the effect. The significance level was set at *p* ≤ 0.05.

Once the model was estimated, the model fit was assessed. When assessing the fit of the theoretical model, different indices were found. These are classified into absolute fit indices and comparative fit indices [14]. Within the absolute fit indices, the most common is the Chi-square/degrees of freedom (χ^2^/gl). Despite being the most common, it is very sensitive to sample size [14]. Values below five show a good fit for this index [14]. Continuing with the comparative fit indices, the most commonly used are the Tucker–Lewis index (TLI), adjusted goodness-of-fit index (AGFI), goodness-of-fit index (CFI) and goodness-of-fit index (GFI) [14]. For these indices, the fit values have to be higher than 0.900 [14,15]. It is also advisable to use other fit indices, such as the root mean squared residuals of approximation (RMSEA). For this, the values should be less than 0.100 [15]. Table 1 shows the fit values for the different indices consulted.

## 3. Results

Table 2 together with Table 3 show the correlational results before and after the intervention programme was implemented.

It was observed that the intervention programme decreased the correlations between pure overt aggression with reactive overt aggression (r = 0.611 *p* ≤ 0.01; r = 0.299 *p* ≤ 0.01), instrumental overt aggression (r = 0.552 *p* ≤ 0.01; r = 0.524 *p* ≤ 0.01), pure relational aggression (r = 0.561 *p* ≤ 0.01; r = 0.394 *p* ≤ 0.01), reactive relational aggression (r = 0.573 *p* ≤ 0.01; r = 0.403 *p* ≤ 0.01) and instrumental relational aggression (r = 0.673 *p* ≤ 0.01; r = 0.566 *p* ≤ 0.01). Next, reactive overt aggression showed another relational decrease with instrumental overt aggression (r = 0.698 *p* ≤ 0.01; r = 0.386 *p* ≤ 0.01), pure relational aggression (r = 0.581 *p* ≤ 0.01; r = 0.386 *p* ≤ 0.01), reactive relational aggression (r = 0.838 *p* ≤ 0.01; r = 0.661 *p* ≤ 0.01) and instrumental relational aggression (r = 0.713 *p* ≤ 0.01; r = 0.629 *p* ≤ 0.01). Overt instrumental aggression showed a correlational decrease with pure relational aggression (r = 0.652 *p* ≤ 0.01; r = 0.594 *p* ≤ 0.01), reactive relational aggression (r = 0.722 *p* ≤ 0.01; r = 0.706 *p* ≤ 0.01) and instrumental relational aggression (r = 0.638 *p* ≤ 0.01; r = 0.613 *p* ≤ 0.01). Likewise, a correlational decrease was obtained from pure relational aggression towards reactive relational aggression (r = 0.583 *p* ≤ 0.01; r = 0.575 *p* ≤ 0.01) and instrumental relational aggression (r = 0.548 *p* ≤ 0.01; r = 0.331 *p* ≤ 0.01). Reactive relational aggression showed a correlational decrease with instrumental relational aggression (r = 0.644 *p* ≤ 0.01; r = 0.587 *p* ≤ 0.01).

**Figure 1:**
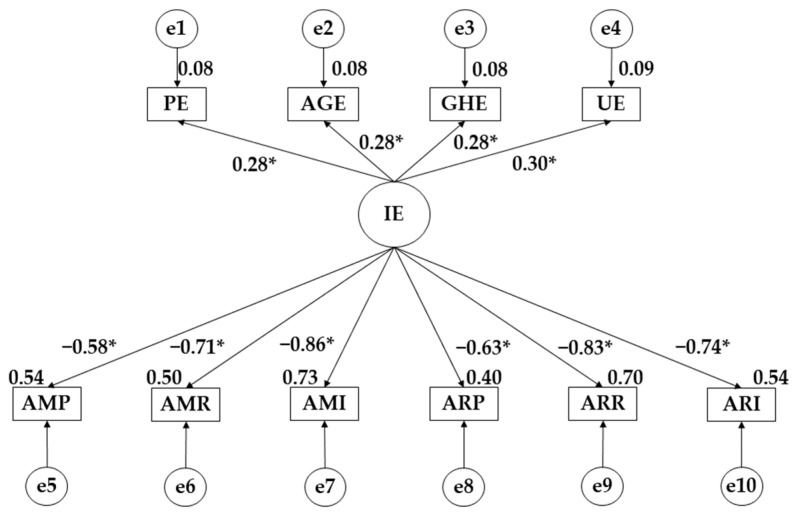
Theoretical model with the pre-test regression weights. Note: * *p* ≤ 0.05 Note: Pure Manifest Aggression (MPA); Manifest Relational Aggression (MRA); Manifest Instrumental Aggression (MIA); Pure Relational Aggression (PRA); Reactive Relational Aggression (RRA); Instrumental Relational Aggression (IRA); Emotional Perception (EP); Emotional Self-management (AGE); Heteroemotional Management (GHE); Emotional Utilisation (UE).

**Figure 2:**
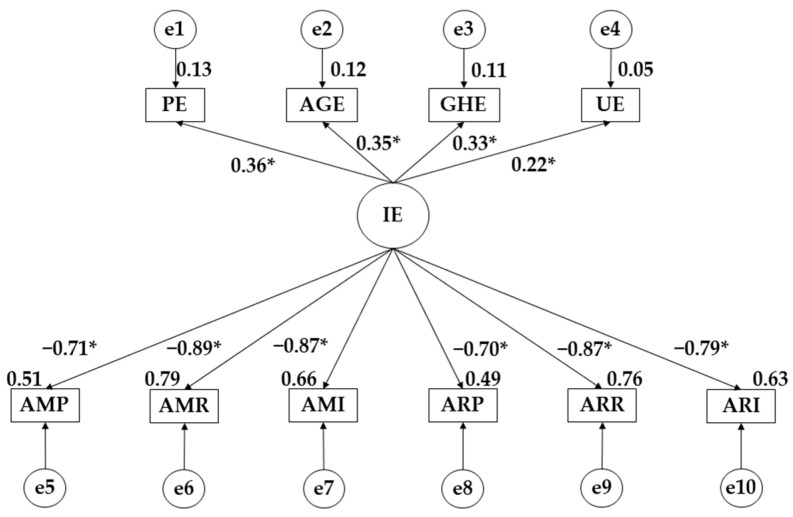
Theoretical model with post-test regression weights. Note: * *p* ≤ 0.05 Note: Pure Manifest Aggression (MPA); Manifest Relational Aggression (MRA); Manifest Instrumental Aggression (MIA); Pure Relational Aggression (PRA); Reactive Relational Aggression (RRA); Instrumental Relational Aggression (IRA).

Figure 1 and Figure 2 together with Table 4 and Table 5 show the exploratory pre-test–post-test analysis.

It was observed that the intervention programme increased the effect of emotional intelligence on emotional self-management (β = 0.281 *p* ≤ 0.05; β = 0.348 *p* ≤ 0.05), heteroemotional management (β = 0.284 *p* ≤ 0.05; β = 0.329 *p* ≤ 0.05) and emotional perception (β = 0.279 *p* ≤ 0.05; β = 0.353 *p* ≤ 0.05). A decrease in the effect of emotional intelligence on emotional utilisation was observed (β = 0.303 *p* ≤ 0.05; β = 0.220 *p* ≤ 0.05). In terms of the effect of emotional intelligence on the different types of violence, an increase in the negative effect was observed for pure overt aggression (β = −0.707 *p* ≤ 0.05; β = −0.888 *p* ≤ 0.05), relational overt aggression (β = −0.579 *p* ≤ 0.05; β = −0.709 *p* ≤0.05), pure relational aggression (β = −0.628 *p* ≤ 0.05; β = −0.704 *p* ≤ 0.05), reactive relational aggression (β = −0.830 *p* ≤ 0.05; β = −0.873 *p* ≤ 0.05), instrumental relational aggression (β = −0.735 *p* ≤ 0.05; β = −0.789 *p* ≤ 0.05) and instrumental overt aggression (β = −0.858 *p* ≤ 0.05; β = −0.869 *p* ≤ 0.05).

## 4. Discussion

This section attempts to compare the results of this study with those of other similar studies. Initially, it was observed that the intervention programme decreased the correlations between the different types of aggressive behaviour. Previous studies have found that sports practice can encourage the appearance of certain emotional states that promote violent behaviour [16]. It was discovered that the motivation or motives for practicing sports favour or disfavour this type of behaviour [17]. Castro-Sánchez et al. [18] state that when practicing sports is based on the achievement of an external goal, emotional states appear that can lead to violent behaviour. When physical/sports practice is oriented towards intrinsic motivation, there is a tendency to reduce negative emotional states [18]. When it comes to violent behaviour, it has been observed that verbal behaviour is the most common in the adolescent population [19]. Studies claim that once verbal aggressive behaviours have been carried out, the next to take place are those involving physical contact [20]. These results suggest that Judo is an effective sport for reducing any type of aggressive behaviour [7,21].

With respect to the exploratory analysis, it was observed that after the application of the intervention programme, the negative links between emotional intelligence and each type of violent behaviour decreased. In view of these findings, it has been stated that Judo, despite being based on grappling actions, is a sport that requires a high level of emotional competence [22]. Although Judo is a contact sport, its philosophical foundations include honour, modesty, respect and self-control [23]. Emotional intelligence is improved through contact sports due to the importance of emotions during their practice [24]. Given these findings, Antoñanzas [25] found that emotional intelligence exerts a buffering effect on aggressive behaviour in the adolescent population. Furthermore, Alvarado et al. [26] and Segura et al. [27] stated that children with higher emotional intelligence tend to reduce the likelihood of aggressive behaviours. Similar research has found that contact sports are very useful for the emotional education of young people [25]. Estévez et al. [27] claim that emotional intelligence exerts a buffering effect on disruptive behaviour [27]. This means that more emotional training reduces violent disruptive behaviour [28,29].

The practical viability of this programme is centred on its compliance with Spanish educational regulations. This regulation states in its text that sports initiation should be carried out through games [30]. Likewise, it is essential to work on interdisciplinarity and to achieve various learning processes simultaneously through play [30]. Through play, the aim is to promote values related to the body, movement and the relationship with the environment [30].

This study is not without its limitations. The sample is only from one school. It would be advisable for future intervention programmes to use more than one school. As a limitation of this research, it is worth highlighting that physical/sports-related activities carried out during extracurricular hours were not taken into account. It should be noted that only one intervention group was used. It would be interesting to repeat this intervention programme by including a design based on a control group and an experimental group. It would also be advisable for future research to carry out a comparative study depending on the grade. It would be useful to carry out an analysis according to the gender of the participants.

## 5. Conclusions

This research shows that a Judo-based intervention programme is effective in improving the effect of emotional intelligence on violent behaviour and in reducing the correlation between different forms of direct and indirect violence. This is why contact sports should be taken into account more in the subject of physical education in order to promote the holistic education of young people.

Based on these results, physical education teachers can take this type of sport into account to improve the behaviours of students when carrying out any physical/sporting activity.

## Figures and Tables

**Table 1:** Fit indices of the theoretical model.

χ^2^/gl	RMSEA	AGFI	GFI	CFI	TLI	NFI
2.859	0.079	0.955	0.958	0.950	0.977	0.984

**Table 2:** Correlational analysis prior to implementation of the intervention programme.

	AMP	AMR	AMI	ARP	ARR	ARI
**AMP**	1					
**AMR**	0.611 **	1				
**AMI**	0.552 **	0.698 **	1			
**ARP**	0.561 **	0.581 **	0.652 **	1		
**ARR**	0.573 **	0.838 **	0.706 **	0.583 **	1	
**ARI**	0.673 **	0.713 **	0.638 **	0.548 **	0.644 **	1
**UE**	0.360 **	0.161 **	0.200 **	0.186 **	0.137 **	0.216 **

Note: ** Correlation is significant at the 0.01 level (bilateral); Note: Pure Manifest Aggression (MPA); Manifest Relational Aggression (MRA); Manifest Instrumental Aggression (MIA); Pure Relational Aggression (PRA); Reactive Relational Aggression (RRA); Instrumental Relational Aggression (IRA).

**Table 3:** Correlational analysis after implementation of the intervention programme.

	AMP	AMR	AMI	ARP	ARR	ARI
**AMP**	1					
**AMR**	0.299 **	1				
**AMI**	0.524 **	0.551 **	1			
**ARP**	0.394 **	0.386 **	0.594 **	1		
**ARR**	0.403 **	0.661 **	0.722 **	0.575 **	1	
**ARI**	0.566 **	0.629 **	0.613 **	0.331 **	0.587 **	1

Note: ** Correlation is significant at the 0.01 level (bilateral); Note: Pure Manifest Aggression (MPA); Manifest Relational Aggression (MRA); Manifest Instrumental Aggression (MIA); Pure Relational Aggression (PRA); Reactive Relational Aggression (RRA); Instrumental Relational Aggression (IRA).

**Table 4:** Standardised regression weights prior to intervention programme implementation.

Direction	Regression Weights				Standardised Regression Weight
	Estimation	Estimation Error	Critical Ratio	*p*	Estimations
**PE ← IE**	1.000				0.279
**AGE ← IE**	0.918	0.309	2.969	0.003	0.281
**GHE ← IE**	−0.727	0.336	−2.161	0.031	0.284
**UE ← IE**	1.359	0.433	3.139	0.002	0.303
**AMP ← IE**	−2.712	0.681	−3.983	0.001	−0.579
**AMR ← IE**	−2.344	0.568	−4.125	0.005	−0.707
**AMI ← IE**	−2.524	0.616	−4.103	0.010	−0.858
**ARP ← IE**	−2.529	0.634	−3.980	0.032	−0.628
**ARR ← IE**	−2.212	0.537	−4.120	0.015	−0.830
**ARI ← IE**	−2.297	0.564	−4.073	0.039	−0.735

Note: Pure Manifest Aggression (MPA); Manifest Relational Aggression (MRA); Manifest Instrumental Aggression (MIA); Pure Relational Aggression (PRA); Reactive Relational Aggression (RRA); Instrumental Relational Aggression (IRA); Emotional Perception (EP); Emotional Self-management (AGE); Heteroemotional Management (GHE); Emotional Utilisation (UE).

**Table 5:** Standardised regression weights after intervention programme implementation.

Direction	Regression Weights				Standardised Regression Weight
	Estimation	Estimation Error	Critical Ratio	*p*	Estimations
**PE ← IE**	1.000				0.353
**AGE ← IE**	0.798	0.350	2.278	0.023	0.348
**GHE ← IE**	−1.089	0.456	−2.389	0.017	0.329
**UE ← IE**	1.219	0.530	2.299	0.021	0.220
**AMP ← IE**	−1.287	0.415	−3.099	0.002	−0.709
**AMR ← IE**	−2.405	0.805	−2.987	0.003	−0.888
**AMI ← IE**	−2.639	0.831	−3.175	0.002	−0.869
**ARP ← IE**	−2.258	0.743	−3.040	0.002	−0.704
**ARR ← IE**	−1.798	0.568	−3.167	0.002	−0.873
**ARI ← IE**	−2.309	0.741	−3.118	0.002	−0.789

Note: Pure Manifest Aggression (MPA); Manifest Relational Aggression (MRA); Manifest Instrumental Aggression (MIA); Pure Relational Aggression (PRA); Reactive Relational Aggression (RRA); Instrumental Relational Aggression (IRA); Emotional Perception (EP); Emotional Self-management (AGE); Heteroemotional Management (GHE); Emotional Utilisation (UE).

## Data Availability

The data used to support the findings of the current study are available from the corresponding author upon request.
